# 24S,25-Epoxycholesterol in mouse and rat brain

**DOI:** 10.1016/j.bbrc.2014.05.012

**Published:** 2014-06-27

**Authors:** Yuchen Wang, Kersti Karu, Anna Meljon, John Turton, Joyce L. Yau, Jonathan R. Seckl, Yuqin Wang, William J. Griffiths

**Affiliations:** a Clinical Laboratory, Jinan Infectious Disease Hospital, Shandong University, Jinan, Shandong, China; b UCL School of Pharmacy, 29-39 Brunswick Square, London WC1N 1AX, UK; c Institute of Mass Spectrometry, College of Medicine, Grove Building, Swansea University, Singleton Park, Swansea SA2 8PP, UK; d Endocrinology Unit, BHF Centre for Cardiovascular Science, The Queen’s Medical Research Institute, University of Edinburgh, 47 Little France Crescent, Edinburgh EH16 4TJ, UK

**Keywords:** HMG-CoA, 3-hydroxy-3-methyl-glutaryl-CoA, APCI, atmospheric pressure chemical ionisation, API, atmospheric pressure ionisation, BBB, blood brain barrier, CNS, central nervous system, Ctx, cortex, CYP or Cyp, cytochrome P450, DDA, data dependent analysis, ER, endoplasmic reticulum, ESI, electrospray ionisation, GC, gas chromatography, GP, Girard P, Insig, insulin-induced gene, LIT, linear ion-trap, LC, liquid chromatography, LXRs or Lxrs, liver X receptors, MS, mass spectrometry or mass spectrum, MS^*n*^, mass spectrometry with multistage fragmentation, MRM, multiple reaction monitoring, RICs, reconstructed ion chromatogram, SCAP, SREBP-cleavage activating protein, SREBP-2, sterol regulatory element-binding protein-2, Vm, ventral midbrain, wt, wild type, 24S,25-Epoxycholesterol, 24S-hydroxycholesterol, 3β,7α-Dihydroxycholest-5-en-24S, 25-epoxide, Brain, Cytochrome P450 7b1

## Abstract

24S,25-Epoxycholesterol is formed in a shunt of the mevalonate pathway that produces cholesterol. It is one of the most potent known activators of the liver X receptors and can inhibit sterol regulatory element-binding protein processing. Until recently analysis of 24S,25-epoxycholesterol at high sensitivity has been precluded by its thermal lability and lack of a strong chromophore. Here we report on the analysis of 24S,25-epoxycholesterol in rodent brain where its level was determined to be of the order of 0.4–1.4 μg/g wet weight in both adult mouse and rat. For comparison the level of 24S-hydroxycholesterol in brain of both rodents was of the order of 20 μg/g, while that of cholesterol in mouse was 10–20 mg/g. By exploiting knockout mice for the enzyme oxysterol 7α-hydroxylase (Cyp7b1) we show that this enzymes is important for the subsequent metabolism of the 24S,25-epoxide.

## Highlights

• 24S,25-Epoxycholesterol identified and quantified in rodent brain.
• Knock out of *Cyp27a1* leads to a decrease in 24S,25-epoxycholesterol.
• Knock out of *Cyp7b1* leads to an increase in 24S,25-epoxycholesterol.
• 24S,25-Epoxycholesterol is metabolised by Cyp7b1 but not Cyp27a1.

## Graphical abstract

**Figure:**
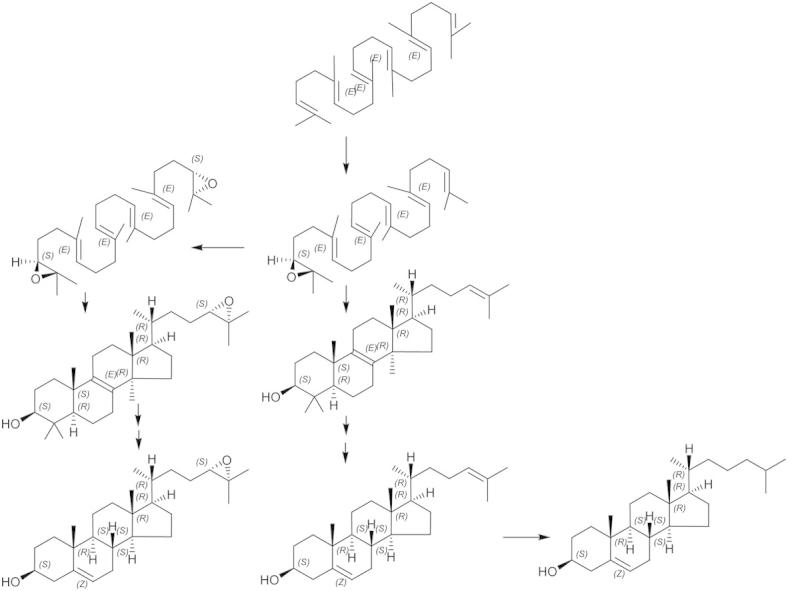

## 1 Introduction

24S,25-Epoxycholesterol (3β-hydroxycholest-5-en-24S,25-epoxide) was first discovered by Nelson et al. in 1981 [1]. It was detected in human liver and found to decrease the activity of 3-hydroxy-3-methyl-glutaryl-CoA (HMG-CoA) reductase, the rate limiting enzyme of the mevalonate pathway leading to cholesterol (cholest-5-en-3β-ol) synthesis [2]. The expressions of enzymes of the mevalonate pathway are regulated by the master transcription factor, sterol regulatory element-binding protein-2 (SREBP-2). In times of cholesterol depletion SREBP-2, synthesised in the endoplasmic reticulum (ER), is escorted by SREBP-cleavage activating protein (SCAP) to the Golgi where it is proteolysed to its active form as a transcription factor. Alternatively, in times of cholesterol wealth, cholesterol binds to SCAP causing a conformational change and inducing SCAP to bind to the anchor protein INSIG (insulin-induced gene) and tethering the SCAP–SREBP-2 complex in the ER, thereby preventing its conversion to an active transcription factor [3]. 24S,25-Epoxycholesterol, and also side-chain hydroxylated cholesterol metabolites (collectively known as oxysterols), can bind to INSIG and similarly tether the SCAP–SREBP-2 complex in the ER and prevent formation of the active transcription factor [4]. In this way cholesterol and oxysterols provide feedback control of cholesterol synthesis. INSIG proteins can also bind to HMG-CoA reductase with the formation of a HMG-CoA reductase – INSIG complex which leads to ubiquitination and degradation of HMG-CoA reductase [3]. Oxysterols induce formation of the HMG-CoA reductase–INSIG complex but cholesterol has no effect. Based on studies by Goldstein and Brown’s group it is tempting to speculate that the oxysterol-bound form of INSIG can form a complex with HMG-CoA reductase just as it can with SCAP and thereby modulate cholesterol synthesis [4].

The capacity of oxysterols to inhibit sterol synthesis in cultured cells lead to the formulation of the oxysterol hypothesis which asserts that the suppressive effect of cholesterol on its own synthesis is mediated by oxysterols [5]. With the discoveries by Goldstein and Brown of cholesterol induced effects on SREBP-2 [3], this hypothesis has been revised to argue that oxysterols play an important role in smoothing cholesterol responses in the short term and providing “fine-tuning” of the acute control of cholesterol homeostasis [6,7].

Oxysterols are formed enzymatically, mostly in cytochrome P450 catalysed (CYP in human, Cyp in rodent) reactions, but can also be formed non-enzymatically [8]. For example, in brain 24S-hydroxycholesterol (cholest-5-en-3β,24S-diol) is abundant as a consequence of 24S-hydroxylation of cholesterol by CYP46A1, while 7α-hydroxycholesterol (cholest-5-en-3β,7α-diol) is a product of liver specific CYP7A1 hydroxylation of cholesterol [9]. Alternatively, 25-hydroxycholesterol (cholest-5-en-3β,25-diol) can be formed enzymatically by cholesterol 25-hydroxylase, which is not a CYP, and by autoxidation in air. Unlike the hydroxycholesterols above, 24S,25-epoxycholesterol is not a product of cholesterol but is formed in parallel to it, and despite its potent biological activity it has been rarely characterised *in vivo*, and little if anything is known of its metabolism.

The central nervous system (CNS) contains about 23% of the body’s cholesterol in adult human and 15% in adult mouse [10]. The blood brain barrier (BBB) isolates the CNS from the circulation, and this dictates that after birth essentially all cholesterol is synthesised *de novo* and *in situ*. Excess cholesterol is exported from brain in the form of 24S-hydroxycholesterol, which unlike cholesterol can traverse the BBB [11]. 24S-Hydroxycholesterol, as discussed above, can also modulate cholesterol synthesis, at least *in vitro* [12]. However, the ability of all cholesterogenic cells to potentially produce 24S,25-epoxycholesterol raises the possibility that this molecule may be equally or more important for the modulation of cholesterol synthesis in brain. Data from Wong et al. [13] indicates that 24S,25-epoxycholesterol can be produced by primary neurons and astrocytes, the latter being the more productive, and that 24S,25-epoxycholesterol synthesised in astrocytes can have downstream effects on gene regulation by neurons. This then begs the question, what are the endogenous levels of 24S,25-epoxycholesterol in brain and how is it metabolised?

**Fig. 1:**
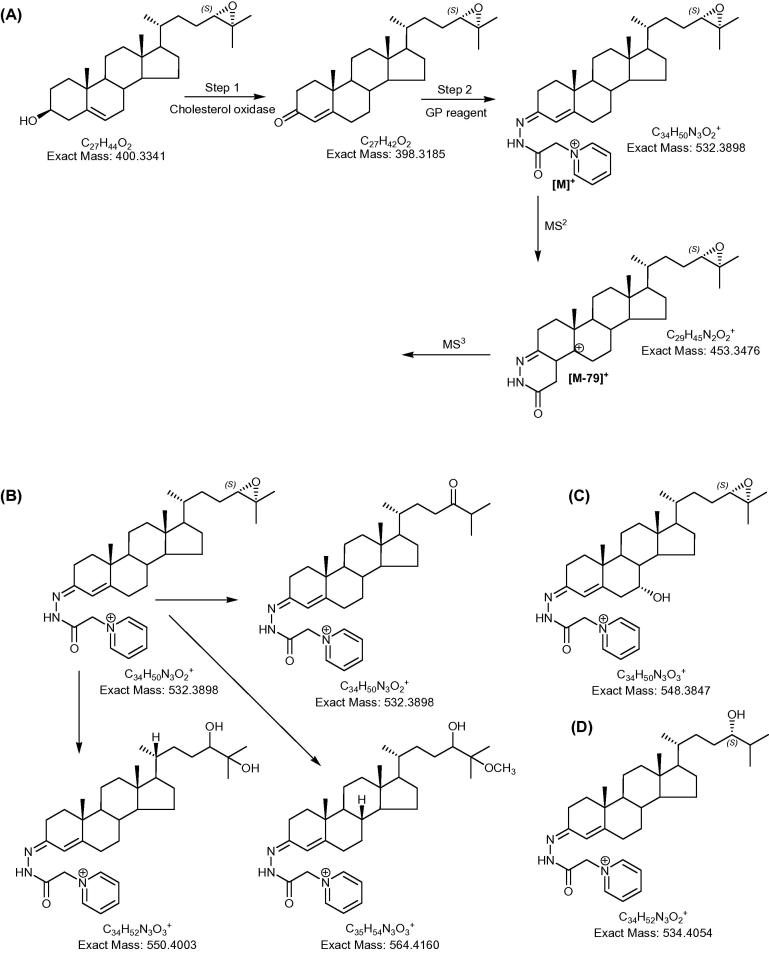
(A) Oxidation with cholesterol oxidase (step 1), followed by derivatisation with the GP reagent (step 2) and fragmentation by mass spectrometry (MS^2^). The reactions are exemplified by 24S,25-epoxycholesterol. (B) Structure of 24S,25-epoxycholesterol its isomer 24-oxocholesterol and hydrolysis and methanolysis products after oxidation/derivatisation. (C) Structure of 3β,7α-dihydroxycholest-5-en-24S,25-epoxide and (D) 24S-hydroxycholesterol following oxidation/derivatisation.

24S,25-Epoxycholesterol is difficult to analyse by gas-chromatography (GC)–mass spectrometry (MS) on account of its thermal lability. As a consequence it is often excluded in sterol profiling experiments based on GC–MS [14,15]. Alternatively, it can be analysed by liquid chromatography (LC)–MS based methods using atmospheric pressure ionisation (API) such as electrospray ionisation (ESI) or atmospheric pressure chemical ionisation (APCI) [13,16,17]. However, sensitivity is limited, requiring application of multiple reaction monitoring (MRM) “scans” to obtain the necessary sensitivity for analysis of biological samples [17]. LC has also been used with UV detection, where oxysterols with a 3β-hydroxy-5-ene structure are converted by cholesterol oxidase to their 3-oxo-4-ene analogues and UV detection is performed for this chromophore at 233 nm (Fig. 1A, Step 1) [18]. Using this LC–UV method the estimated lower limit of detection of oxysterols including 24S,25-epoxycholesterol was about 2 ng. This degree of sensitivity allowed the detection and quantification of 24S,25-epoxycholesterol in rat liver. For comparison, McDonald et al. determined the on-column detection limit for 24S,25-epoxycholesterol using LC–ESI-MRM to be 20 fmol (8 pg) and was able to identify this oxysterol in mouse brain [17]. In an effort to improve sensitivity of oxysterol analysis by LC–API-MS, we and others have developed methods based on chemical derivatisation to increase analyte signal [19–21]. We have exploited oxidation of oxysterol 3β-hydroxy-5-ene groups to 3-oxo-4-enes cf. [18], followed by derivatisation with charge carrying tags i.e., with Girard P (GP) hydrazine (Fig. 1A), and LC–ESI-MS analysis [20,21]. By performing analysis on an ion-trap it was possible to perform multiple stages of fragmentation (MS*^n^*) which provides structural information to compliment molecular weight information of the MS scan. On-column detection limits for recording full scan MS, MS^2^ and MS^3^ spectra were of the order of 1.5 pg. Here we report on the identification and quantification of 24S,25-epoxycholesterol in adult mouse and rat brain and the determination of its abundance in comparison to the major brain derived oxysterol, 24S-hydroxycholesterol. We then investigate the metabolism of 24S,25-epoxycholesterol by exploiting the oxysterol 7α-hydroxylase knockout mouse (*Cyp7b1*^−/−^).

## 2 Methods

### 2.1 Animals

Rat brain samples were from dissected from 15-week female Sprague Dawley animals (Harlan UK). Male *Cyp7b1*^−/−^ mouse brain was from animals generated at the University of Edinburgh. Male mice, 13 months and 23 months, homozygous for targeted disruption of the *Cyp7b1* gene congenic on the C57BL/6 genetic background (>15 generations backcrossed to C57BL/6) and wild-type (wt) littermate controls were generated from *Cyp7b1*^−/+^ crosses [22]. Male *Cyp27a1*^−/−^ mouse (3 months) tissue was purchased from The Jackson Laboratory (ME, USA) strain B6.129-Cyp27a1^tm1Elt^/J [23]. The *Cyp27a1*^−/−^ colony was backcrossed to C57BL/6J inbred mice for approximately 12 generations by the donating investigator [23] prior to sending to The Jackson Laboratory Repository. Upon arrival, mice were bred to C57BL/6J inbred mice for at least one generation to establish the colony. Wild type animals from the colony were used as controls. Tissue sampling was performed under the aegis of the UK Scientific Procedures (Animals) Act, 1986.

### 2.2 Extraction of oxysterols and analysis

Adult rat or mouse brain (100–200 mg wet weight) was homogenised in ethanol and an oxysterol rich fraction isolated from more non-polar sterols (mostly cholesterol) by solid phase extraction as described by Karu et al. [20] and by Meljon et al. [21]. The oxysterol fraction was then oxidised with cholesterol oxidase and derivatised with GP reagent [20,21] (Fig. 1A). The oxidised/derivatised oxysterols were then analysed by LC–ES-MS*^n^* utilising an LTQ-Orbitrap (Thermo Scientific, Hemel Hempstead, UK) hybrid linear ion-trap (LIT)–Orbitrap mass spectrometer [20,21]. The instrument was operated in a data dependent analysis (DDA) mode where MS scans were recorded in the Orbitrap at 30,000 resolution (full width at half maximum height definition) and if an ion corresponding to oxidised/derivatised 24S,25-epoxycholesterol (*m*/*z* 532.3898 or adducts thereof *m*/*z* 550.4003 and 564.4160) or 24S-hydroxycholesterol (*m*/*z* 534.4054) was detected, MS^2^ was performed in the LIT portion of the instrument (Fig. 1). GP derivatives give a characteristic loss of 79.04 Da and if this was observed in the MS^2^ spectrum, MS^3^ was performed in the LIT on the [M−79]^+^ ion (Fig. 1A). The LTQ-Orbitrap was operated so that MS^2^ or MS^3^ events were performed in the LIT at the same time as the MS scan was performed in the Orbitrap. The cycle of events was continually repeated over the course of the LC run [20,21]. Alternatively, the instrument was operated in a MRM-like mode where the defined transitions [M]^+ ^→ [M−79]^+^ → were monitored in an MS^3^ event at the same time as a mass scan was performed in the Orbitrap [24].

Previous studies have shown that 3β-hydroxy-5-ene sterols following oxidation and derivatisation with the GP reagent give a similar response upon LC–ES–MS analysis, this then allows their relative quantification from chromatographic peak areas [20,25]. The lability of the epoxide group is such that 24S,25-epoxycholesterol gives four components following oxidation/derivatisation. The expected [M]^+^ ion at *m*/*z* 532.3898 corresponding to the oxidised/derivatised epoxide and to its 24-oxo isomer and to “adduct” ions at *m*/*z* 550.4003 and *m*/*z* 564.4160 (Fig. 1B). These latter ions correspond to the [M]^+^ ions of the 24,25-diol and the 24-hydroxy-25-methylether (or 25-hydroxy-24-methylether) which are products of acid calalysed hydrolysis and methanolysis of the epoxide, respectively. However, as each component gives a similar LC–ES-MS response their peak areas can be combined to provide quantitative information for the parent epoxide.

## 3 Results

**Fig. 2:**
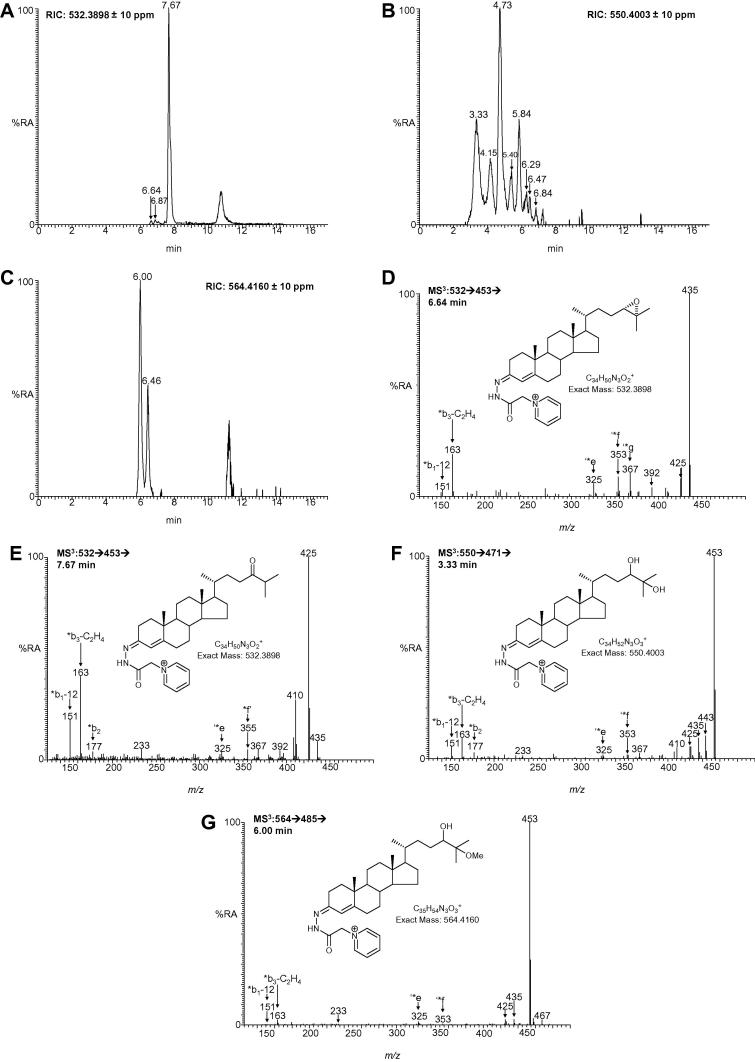
LC–ESI-MS*^n^* analysis of 24S,25-epoxycholesterol in brain of wt mouse following treatment with cholesterol oxidase and derivatisation with GP-reagent. Reconstructed ion chromatograms (RICs) of (A) *m*/*z* 532.3898, (B) 550.4003 and 564.4160. MS^3^ fragmentation spectra of the peaks eluting at (D) 6.64 min and (E) 7.67 min in the RIC of *m*/*z* 532.3898, at (F) 3.33 min in the RIC of *m*/*z* 550.4003 and at (G) 6.00 min in the RIC of *m*/*z* 564.4160. Structures of the oxidised/derivatised molecules are shown as insets. Fragment ions are described in Karu et al. [20]. *Syn* and *anti* forms of the derivatives are formed resulting in duplicate peak for 24S,25-epoxycholesterol at 6.64 and 6.87 in (A), for 24,25-dihydroxycholesterol at 3.33 and 4.15 min in (B) and for 24-hydroxy-25-methoxycholesterol at 6.00 and 6.46 min in (C). Other peaks in (B) correspond to oxidised/derivatised unresolved 7α,24- and 7α,25-dihydroxycholesterols at 5.40 min, 7α,26-dihydroxycholesterol at 5.84 and 6.84 min, 7α,25-dihydroxycholesterol at 6.29 min and 7α,24-dihydroxycholesterol at 6.47 min.

In an initial LC–ESI-MS*^n^* study with female rat brain [M]^+^ ions corresponding to oxidised/derivatised 24S,25-epoxycholesterol, its isomer 24-oxocholesterol, and hydrolysis and methanolysis products were observed. Their identification was confirmed by MS^2^, MS^3^ and by reference to authentic standards. The resultant concentration of 24S,25-epoxycholesterol was determined to be 0.53 ± 0.46 μg/g (mean ± SD, *n* = 4) wet weight. More detailed studies were then performed on male mice. Shown in Fig. 2 are reconstructed ion chromatograms (RICs) for *m*/*z* 532.3898, 550.4003 and 564.4160 corresponding to 24S,25-epoxycholesterol derived [M]^+^ ions from wt adult mouse brain. Comparison of chromatographic retention times and MS^2^ and MS^3^ spectra to those of authentic standards confirmed the identification of the relevant peaks (Fig. 2). In male wt mice the level of 24S,25-epoxycholesterol varied from 0.44 to 1.32 μg/g depending on age and exact genotype (Table S1). For comparison, the level of 24S-hydroxycholesterol ranged from 16.82 to 25.96 μg/g in wt mice, while that of cholesterol varied from 10.60 to 16.90 mg/g.

**Fig. 3:**
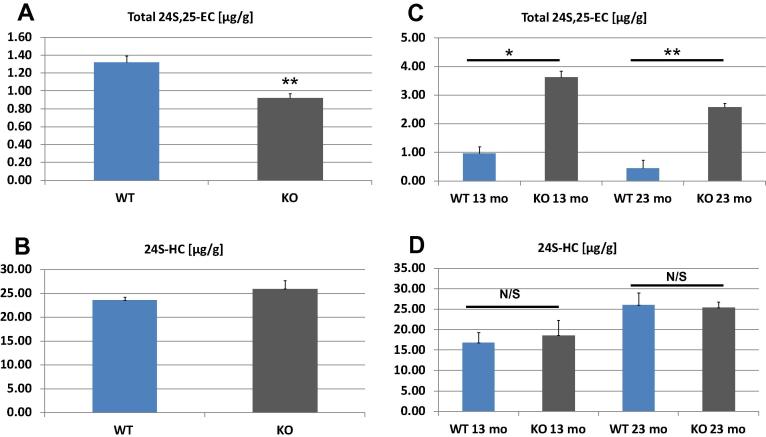
Levels of (A) 24S,25-epoxycholesterol and (B) 24S-hydroxycholesterol in *Cyp27a1*^−/−^ and wt control mouse brain (3 months, n = 3) and in (C,D) *Cyp7b1*^−/−^ and wt control mouse brain (13 months, n = 3; 23 months, *n* = 4). ^∗^*p* < 0.05, ^∗∗^*p* < 0.01 by Student’s *t*-test.

To investigate the route of metabolism of 24S,25-epoxycholesterol in brain we utilised two different knockout mouse models, *Cyp27a1*^−/−^ and *Cyp7b1*^−/−^ where the expression of the Cyp enzyme sterol 27-hydroxylase (Cyp27a1) or oxysterol 7α-hydroxylase (Cyp7b1) is eliminated respectively. The level of the epoxide in the *Cyp27a1*^−/−^ mouse was a little lower than the control wt (0.92 ± 0.05 μg/g cf. 1.32 ± 0.07 μg/g, *n* = 3, *p* < 0.01, Fig. 3A) while the levels of 24S-hydroxycholesterol (25.92 ± 1.73 μg/g cf. 23.55 ± 0.65 μg/g, Fig. 3B) and of cholesterol (16.98 ± 0.96 mg/g cf. 16.90 ± 0.29 mg/g) were essentially unchanged between the knockout and wt control mice. In contrast, the level of 24S,25-epoxycholesterol was greatly elevated in the *Cyp7b1*^−/−^ mice compared to the wt (at 13 months 3.62 ± 0.22 μg/g cf. 0.96 ± 0.24 μg/g, *n* = 3, *p* < 0.05; 23 months 2.58 ± 0.15 μg/g cf. 0.44 ± 0.30 μg/g, *n* = 4, *p* < 0.01, Fig. 3C) while again the levels of neither 24S-hydroxycholesterol (at 13 months 18.58 ± 3.73 μg/g cf. 16.82 ± 2.55 μg/g; 23 months 25.34 ± 1.40 μg/g cf. 25.96 ± 3.00 μg/g, Fig. 3D) nor cholesterol (13 months 10.33 ± 2.02 mg/g cf. 10.60 ± 1.43 mg/g; 23 months 16.85 ± 0.59 mg/g cf. 15.81 ± 0.65 mg/g) differed between the knockout mice and wt controls.

## 4 Discussion

Until recently suitable methods have not been available for the analysis of 24S,25-epoxycholesterol in brain. We performed one previous study in which we measured the quantity of the epoxide in 15 week old adult female mice and the levels determined were similar to those measured hear (0.64 μg/g) [24]. Interestingly, in that study we also analysed the epoxide level in cholesterol 24-hydroxylase knockout mice (*Cyp46a1*^−/−^) and found that the amount of epoxide fell to 0.12 μg/g, while that of cholesterol was constant (16 mg/g). As expected the amount of 24S-hydroxycholesterol in brain of the *Cyp46a1*^−/−^ mice was at trace levels (0.02 μg/g cf. 27.91 μg/g in wt). We explained the low levels of 24S,25-epoxycholesterol in the *Cyp46a1*^−/−^ mice by a reduced synthesis, paralleling that of cholesterol which falls to counteract the effect of the elimination of its export route via 24S-hydroxylation. Although, 24S-hydroxylation by Cyp46a1 represents the major route for cholesterol removal from brain, a minor amount may be eliminated through (25R)26-hydroxylation of cholesterol by Cyp27a1, and subsequent oxidation to 7α-hydroxy-3-oxocholest-(25R)-4-en-26-oic acid which crosses the BBB and enters the circulation [26]. Note that according to current nomenclature Cyp27a1 introduces hydroxylation at C-26 generating R stereochemistry at C-25 [27]. Thus, as the cholesterol and 24S-hydroxycholesterol levels are unchanged in the wt and *Cyp27a1*^−/−^ mice, cholesterol homeostasis is achieved through its reduced synthesis and hence that of the 24S,25-epoxide. The 24S,25-epoxide is formed via a shunt of the Bloch arm of the mevalonate pathway whose penultimate member is desmosterol. Studies by others find that desmosterol is reduced in brain of *Cyp27a1*^−/−^ mice, but surprisingly sterols of the Kandutsch-Russell arm of the pathway are elevated [28].

The two major routes of oxysterol metabolism are via 7α- and (25R)26-hydroxylation through Cyp7b1 (Cyp39a1 in the case of 24S-hydroxycholesterol) and Cyp27a1, respectively [9]. As detailed above knockout of *Cyp27a1* results in a small decrease in the level of 24S,25-epoxycholesterol. In contract knockout of *Cyp7b1* results in a significant increase of the epoxide, strongly indicating that it represents a substrate of this enzyme. When appropriate RICs were generated for 3β,7α-dihydroxycholest-5-en-24S,25-epoxide we failed to find any components giving fragmentation spectra compatible with this structure in any of our mouse brain samples studied, presumably because abundance of the metabolite was below the detection limit. Unfortunately, the authentic standard of 3β,7α-dihydroxycholest-5-en-24S,25-epoxide is not currently commercially available.

It is also of interest to compare the levels of 24S,25-epoxycholesterol in adult (0.4–1.4 μg/g) to that found in the newborn mouse (1.12 μg/g) [21] and in developing mouse brain (E11.5, ventral midbrain, Vm, 0.39 μg/g; cortex, Ctx, 0.33 μg/g) [29,30]. For reference, the levels of 24S-hydroxycholesterol were measured to be 0.51 μg/g in newborn mouse brain and 0.03 μg/g in Vm and Ctx of the developing brain. Although the absolute levels of 24S,25-epoxycholesterol are quite similar in the adult, new born and developing mouse brain, the ratios of 24S,25-epoxycholesterol to 24S-hydroxycholesterol vary by almost 3 orders of magnitude (adult ∼1:60–1:20; newborn ∼2:1, foetus ∼10:1). This is a striking difference, but can be reconciled with the differing routes of formation of these oxysterols. 24S,25-Epoxycholesterol is formed via a shunt of the mevalonate pathway in parallel to cholesterol, while 24S-hydroxycholesterol is formed from cholesterol. In mouse embryonic development the foetal brain becomes the major source of its own cholesterol at E10–E11 [31], thus, at E11 the mouse brain is actively synthesising cholesterol and 24S,25-epoxycholesterol. On the other hand, 24S-hydroxycholesterol is a metabolic product of cholesterol itself, and in developing brain Cyp46a1 expression is low until E18, thereby conserving newly synthesised cholesterol for utilisation by plasma membranes [31]. In contrast, in adult mouse brain where Cyp46a1 is fully expressed in neurons, about half of the newly synthesised cholesterol is converted to 24S-hydroxycholesterol [10], thereby accounting for its appreciable level in brain.

These results are significant when the role of oxysterols in controlling cholesterol levels is considered. Although 24S-hydroxycholesterol suppresses the processing of SREBP-2 and also acts as a ligand to the liver X receptors (Lxrs) *in vitro* [12], its low level in comparison to 24S,25-epoxycholesterol in developing brain questions the relative importance of these two oxysterols in foetal brain. Further, in addition to excreting cholesterol from brain more slowly, *Cyp46a1* knock-out mice synthesise cholesterol via the mevalonate pathway at a reduced rate [32], this argues against a role for 24S-hydroxycholesterol as a major regulator of cholesterol syntheses, but would be consistent with 24S,25-epoxycholesterol playing a role in “fine tuning” the rate of cholesterol synthesis. It should be noted that 24S,25-epoxycholesterol has a further role in the developing midbrain, where through activation of Lxrs it regulates dopaminergic neurogenesis [30].

In conclusion, here we report the levels of 24S,25-epoxycholesterol in rodent brain. Its elevated level in the *Cyp7b1*^−/−^ mouse strongly suggests that it is a substrate for the Cyp7b1 enzyme.

## Appendix A Supplementary data

Supplementary Table S1 24S,25-Epoxycholesterol and 24S-hydroxycholesterol in mouse brain.
