# Supplementary material for: 24S,25-Epoxycholesterol in mouse and rat brain
Source: Biochem Biophys Res Commun. 2014 Jun 27;449(2):229–34. doi: 10.1016/j.bbrc.2014.05.012 (PMC4053837; doi:10.1016/j.bbrc.2014.05.012)
Supplement: Supplementary Table S1 — 24S,25-Epoxycholesterol and 24S-hydroxycholesterol in mouse brain. [file mmc1.docx]

**Supplementary Material**

Table S1. 24S,25-Epoxycholesterol and 24S-hydroxycholesterol in mouse brain

|  | *Male adult mouse brain^a^* | | | | *Female adult mouse brain^b^*  *2 ¾ mo, n = 2* | | *Newborn mouse brain^c^, n =6* | *Embryonic mouse brain^d,e^* |
| --- | --- | --- | --- | --- | --- | --- | --- | --- |
| Sterol  (Systematic Name) | Age (mo),  n of mice | wild type (μg/g)  mean ± SD | *Cyp27a1^-/-^* (μg/g)  mean ± SD | *Cyp7b1^-/-^* (μg/g)  mean ± SD | wild type  (μg/g)  mean ± SD | *Cyp46a1-/-*  (μg/g)  mean ± SD | wild type  (μg/g)  mean ± SD | mean (Vm + Ctx)  (μg/g) |
| Cholesterol  (Cholest-5-en-3β-ol) | 3, n=3  13, n=3  23, n=4 | 16900±290  10600±1430  158100±650 | 16980±960  --  -- | --  10330±2020  16850±590 | 16000 | 16000 | 2221.30±31.68 | 1200 |
| Total 24S,25-Epoxycholesterol  (3β-Hydroxycholest-5-en-24S,25-epoxide) | 3, n=3  13, n=3  23, n=4 | 1.32±0.07  0.96±0.24  0.44±0.30 | 0.92±0.05  --  -- | --  3.62±0.22  2.58±0.15 | 0.64±0.02 | 0.12±0.04 | 1.12±0.30 | 0.36 |
| 24S-Hydroxycholesterol  (Cholest-5-en-3β, 24S-diol) | 3, n=3  13, n=3  23, n=4 | 23.55±0.65  16.82±2.55  25.96±3.00 | 25.92±1.73  --  -- | --  18.58±3.73  25.34±1.40 | 27.91±0.73 | 0.02±0.00 | 0.51±0.08 | 0.03 |

^a^Data from present work.

^b^Data from [24]

^c^Data from [21]

^d^Data from [29]

^e^Data from [30]
